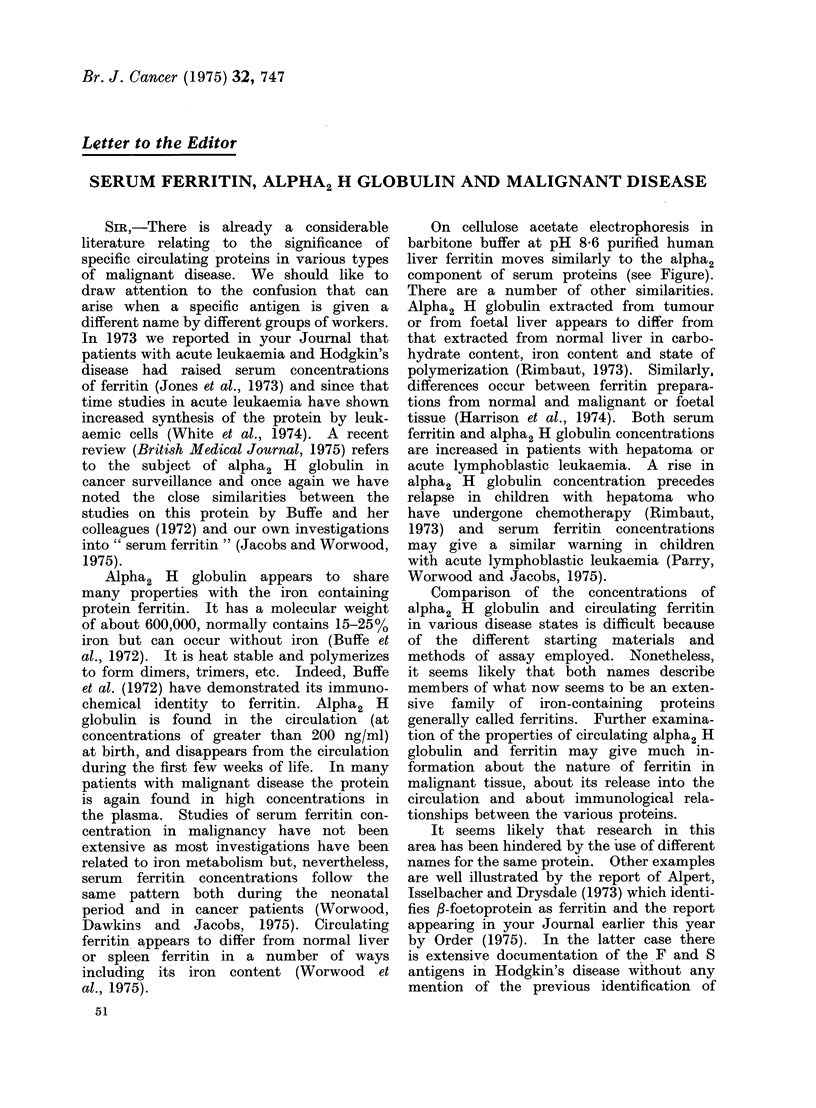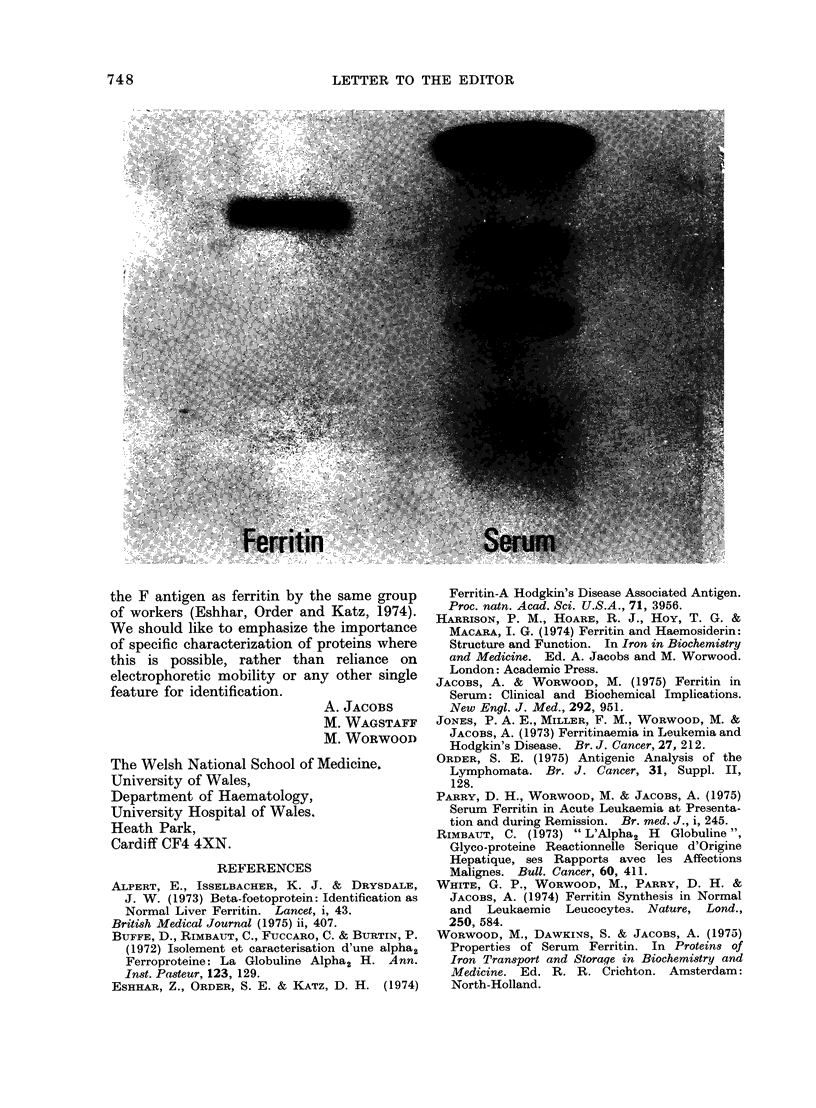# Letter: Serum ferritin, alpha2 H globulin and malignant disease.

**DOI:** 10.1038/bjc.1975.288

**Published:** 1975-12

**Authors:** A. Jacobs, M. Wagstaff, M. Worwood

## Abstract

**Images:**


*Br. J. Cancer* (1975) **32**, 747

### *Letter to the Editor*

### SERUM FERRITIN, ALPHA$_2$ H GLOBULIN AND MALIGNANT DISEASE

SIR,—There is already a considerable literature relating to the significance of specific circulating proteins in various types of malignant disease. We should like to draw attention to the confusion that can arise when a specific antigen is given a different name by different groups of workers. In 1973 we reported in your Journal that patients with acute leukaemia and Hodgkin's disease had raised serum concentrations of ferritin (Jones *et al.*, 1973) and since that time studies in acute leukaemia have shown increased synthesis of the protein by leukaemic cells (White *et al.*, 1974). A recent review (*British Medical Journal*, 1975) refers to the subject of alpha$_2$ H globulin in cancer surveillance and once again we have noted the close similarities between the studies on this protein by Buffe and her colleagues (1972) and our own investigations into " serum ferritin " (Jacobs and Worwood, 1975).

Alpha$_2$ H globulin appears to share many properties with the iron containing protein ferritin. It has a molecular weight of about 600,000, normally contains 15–25% iron but can occur without iron (Buffe *et al.*, 1972). It is heat stable and polymerizes to form dimers, trimers, etc. Indeed, Buffe *et al.* (1972) have demonstrated its immunochemical identity to ferritin. Alpha$_2$ H globulin is found in the circulation (at concentrations of greater than 200 ng/ml) at birth, and disappears from the circulation during the first few weeks of life. In many patients with malignant disease the protein is again found in high concentrations in the plasma. Studies of serum ferritin concentration in malignancy have not been extensive as most investigations have been related to iron metabolism but, nevertheless, serum ferritin concentrations follow the same pattern both during the neonatal period and in cancer patients (Worwood, Dawkins and Jacobs, 1975). Circulating ferritin appears to differ from normal liver or spleen ferritin in a number of ways including its iron content (Worwood *et al.*, 1975).

On cellulose acetate electrophoresis in barbitone buffer at pH 8·6 purified human liver ferritin moves similarly to the alpha$_2$ component of serum proteins (see Figure). There are a number of other similarities. Alpha$_2$ H globulin extracted from tumour or from foetal liver appears to differ from that extracted from normal liver in carbohydrate content, iron content and state of polymerization (Rimbaut, 1973). Similarly, differences occur between ferritin preparations from normal and malignant or foetal tissue (Harrison *et al.*, 1974). Both serum ferritin and alpha$_2$ H globulin concentrations are increased in patients with hepatoma or acute lymphoblastic leukaemia. A rise in alpha$_2$ H globulin concentration precedes relapse in children with hepatoma who have undergone chemotherapy (Rimbaut, 1973) and serum ferritin concentrations may give a similar warning in children with acute lymphoblastic leukaemia (Parry, Worwood and Jacobs, 1975).

Comparison of the concentrations of alpha$_2$ H globulin and circulating ferritin in various disease states is difficult because of the different starting materials and methods of assay employed. Nonetheless, it seems likely that both names describe members of what now seems to be an extensive family of iron-containing proteins generally called ferritins. Further examination of the properties of circulating alpha$_2$ H globulin and ferritin may give much information about the nature of ferritin in malignant tissue, about its release into the circulation and about immunological relationships between the various proteins.

It seems likely that research in this area has been hindered by the use of different names for the same protein. Other examples are well illustrated by the report of Alpert, Isselbacher and Drysdale (1973) which identifies $\beta$-foetoprotein as ferritin and the report appearing in your Journal earlier this year by Order (1975). In the latter case there is extensive documentation of the F and S antigens in Hodgkin's disease without any mention of the previous identification of the F antigen as ferritin by the same group of workers (Eshhar, Order and Katz, 1974). We should like to emphasize the importance of specific characterization of proteins where this is possible, rather than reliance on electrophoretic mobility or any other single feature for identification.

A. JACOBS
M. WAGSTAFF
M. WORWOOD

The Welsh National School of Medicine.
University of Wales,
Department of Haematology,
University Hospital of Wales.
Heath Park,
Cardiff CF4 4XN.

REFERENCES

ALPERT, E., ISSELBACHER, K. J. & DRYSDALE, J. W. (1973) Beta-foetoprotein: Identification as Normal Liver Ferritin. *Lancet*, i, 43.

*British Medical Journal* (1975) ii, 407.

BUFFE, D., RIMBAUT, C., FUCCARO, C. & BURTIN, P. (1972) Isolement et caracterisation d'une alpha$_2$ Ferroproteine: La Globuline Alpha$_2$ H. *Ann. Inst. Pasteur*, **123**, 129.

ESHHAR, Z., ORDER, S. E. & KATZ, D. H. (1974) Ferritin-A Hodgkin's Disease Associated Antigen. *Proc. natn. Acad. Sci. U.S.A.*, **71**, 3956.

HARRISON, P. M., HOARE, R. J., HOY, T. G. & MACARA, I. G. (1974) Ferritin and Haemosiderin: Structure and Function. In *Iron in Biochemistry and Medicine*. Ed. A. Jacobs and M. Worwood. London: Academic Press.

JACOBS, A. & WORWOOD, M. (1975) Ferritin in Serum: Clinical and Biochemical Implications. *New Engl. J. Med.*, **292**, 951.

JONES, P. A. E., MILLER, F. M., WORWOOD, M. & JACOBS, A. (1973) Ferritinaemia in Leukemia and Hodgkin's Disease. *Br. J. Cancer*, **27**, 212.

ORDER, S. E. (1975) Antigenic Analysis of the Lymphomata. *Br. J. Cancer*, **31**, Suppl. II, 128.

PARRY, D. H., WORWOOD, M. & JACOBS, A. (1975) Serum Ferritin in Acute Leukaemia at Presentation and during Remission. *Br. med. J.*, i, 245.

RIMBAUT, C. (1973) " L'Alpha$_2$ H Globuline ", Glyco-proteine Reactionnelle Serique d'Origine Hepatique, ses Rapports avec les Affections Malignes. *Bull. Cancer*, **60**, 411.

WHITE, G. P., WORWOOD, M., PARRY, D. H. & JACOBS, A. (1974) Ferritin Synthesis in Normal and Leukaemic Leucocytes. *Nature, Lond.*, **250**, 584.

WORWOOD, M., DAWKINS, S. & JACOBS, A. (1975) Properties of Serum Ferritin. In *Proteins of Iron Transport and Storage in Biochemistry and Medicine*. Ed. R. R. Crichton. Amsterdam: North-Holland.